# Spatial and temporal evaluation of iodine uptake and radiodensity in meniscus tissue using contrast-enhanced micro-CT

**DOI:** 10.1016/j.heliyon.2024.e41080

**Published:** 2024-12-07

**Authors:** Federica Orellana, Alberto Grassi, Katja M. Nuss, Peter Wahl, Antonia Neels, Stefano Zaffagnini, Annapaola Parrilli

**Affiliations:** a Empa – Swiss Federal Laboratories for Materials Science and Technology, 8600, Dübendorf, Switzerland; b Department of Chemistry, University of Fribourg, 1700, Fribourg, Switzerland; c IRCCS - Rizzoli Orthopaedic Institute, 40136, Bologna, Italy; d Musculoskeletal Research Unit (MSRU), Vetsuisse Faculty, University of Zurich, 8057, Zurich, Switzerland; e Faculty of Medicine, University of Bern, 3008, Bern, Switzerland; f Division of Orthopaedics and Traumatology, Cantonal Hospital Winterthur, 8401, Winterthur, Switzerland

**Keywords:** Iodine, microCT, Meniscus, Contrast agent, Contrast-enhanced micro-CT

## Abstract

**Rationale and objective:**

The visualization of soft tissues, like the meniscus, through X-ray micro-computed tomography (micro-CT), requires the use of contrast agents (CAs). While other studies have investigated CA diffusion in fibrocartilagineous tissues, this work aimed to optimize iodine staining protocols for meniscal tissue that improve their visualization by micro-CT. Specific objectives included evaluating the diffusion of CAs within meniscal samples over time, assessing volume changes due to staining, and identifying the iodine ions absorbed by the tissue.

**Materials and methods:**

Water-based and PBS-based Lugol solutions (KI_3_) were used to stain sheep and pig menisci for 24 days. Samples were scanned using micro-CT at different time points (0, 1, 4, 8, 12, 16, 20, and 24 days) to monitor CA diffusion and volume changes. Micro-CT provided three-dimensional (3D) visualization of iodine distribution and quantification of volume changes and radiodensity in the menisci. Additionally, UV–visible spectroscopy (UV–vis) analyses were performed to determine the uptake of iodine ions by the meniscus.

**Results:**

Results indicated volumetric shrinkage and increased radiodensity within the first days of staining, with diffusion primarily occurring from the periphery of the meniscus. UV–visible spectroscopy identified two iodide ions in the CA solution (I^−^ and I_3_^−^) and revealed a preferential absorption of the triiodide ion (I_3_^−^).

**Conclusion:**

This study demonstrated the utility of iodine-based CAs and micro-CT technique for visualizing and investigating the spatial and temporal iodine diffusion within the meniscal tissue of sheep and pigs. The findings of this study have important implications for using iodine-based CAs in imaging analyses of the meniscus and offer potentially valuable insights into the diffusion patterns of iodine in fibrocartilagineous tissues.

## Graphical abstract

**Figure:**
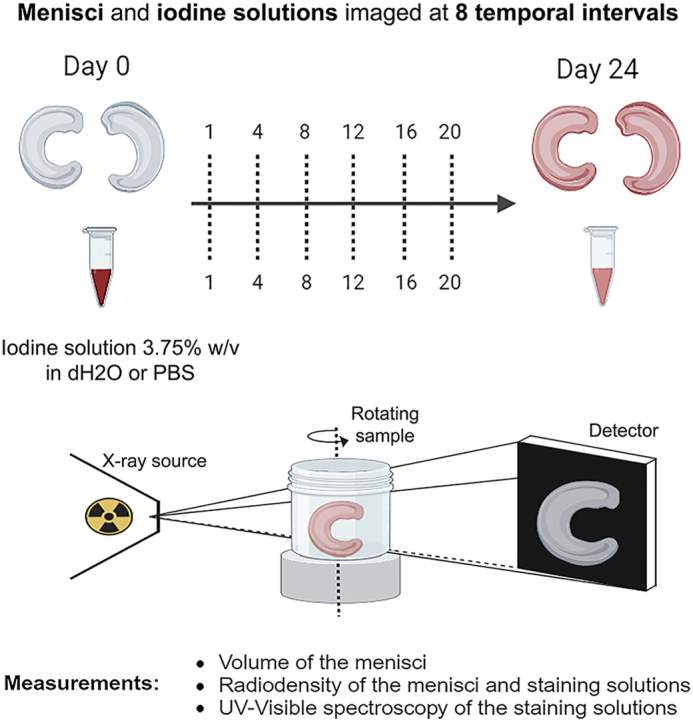

## 1 Introduction

The menisci are a pair of wedge-shaped fibrocartilaginous semilunar structures within the knee joint, located between the femoral condyles and the tibial plateau, improving the incongruence between the cartilaginous surfaces with their concave structure [1,2]. The menisci have a role in load transmission, shock absorption, stability, nutrition, joint lubrication, and proprioception [[3], [4], [5]]. Meniscal degeneration or injury can lead to significant functional impairment and contribute to the development of osteoarthritis (OA), highlighting the importance of understanding meniscal biology and pathology [[6], [7], [8]]. The menisci are composed of a dense extracellular matrix (ECM), primarily comprising water (72 %) and collagen (22 %), with the remaining dry weight attributed to proteoglycans (PGs), non-collagenous proteins, and glycoproteins [4,8].

Among the imaging techniques available for 3D visualization of tissues, X-ray micro-computed tomography (micro-CT) is a non-destructive method capable of exploring the internal microstructure of materials and generating three-dimensional (3D) models [[9], [10], [11], [12]]. It is particularly valuable for imaging high-attenuating structures like bone. However, the visualization of soft tissues remains challenging due to their inherently low X-ray contrast. Therefore, contrast enhancement techniques, such as the use of contrast agents (CAs), are essential for differentiation of the various structures within such samples. Several recent studies have compared various CAs for the visualization of soft tissues [[13], [14], [15]]. Various staining methods, such as iodine-based solutions, phosphotungstic acid (PTA), and osmium tetroxide, have been tested as CAs to observe the development of chicken embryos, distinguishing different organs, and to compare the penetration of the CAs [16]. Iodine is a versatile CA that can be used to enhance the radiodensity of any tissue, as it is known to form complexes with the helical coil structure of the polysaccharide glycogen and its plant counterpart, starch, without bonding specifically to any other cellular or extracellular components [14,[17], [18], [19], [20]]. For tissue penetration of CAs in large samples, only potassium iodide (KI) stained the whole tissue within a short time [13,16]. Lugol (KI_3_) solution is among the anionic iodine-based solutions frequently used as CAs. It is composed of potassium iodide (KI) and iodine (I_2_) in a 2:1 ratio, dissolved in water or ethanol, KI serving to increase the solubility of I_2_. In solution, an equilibrium is established between I_2_ and iodide ion (I^−^), and the resulting triiodide ion (I_3_^−^) [21]:

I_2_ + I^−^ ⇋ I_3_^−^

Within cartilaginous and fibrocartilaginous tissues, iodine distribution occurs mainly by diffusion and is influenced by ECM biochemical constituents [[22], [23], [24]]. PGs, being negatively charged, change the transport characteristics of anionic CAs into the tissue. Indeed, iodine distribution at equilibrium is significantly higher in the meniscus than in cartilage, a result that correlates with the lower content of PGs in the meniscus compared with that in cartilage [25]. Additionally, polar interactions can occur between anionic CAs and polar functional groups, such as –OH and –NH, present in collagen and other ECM components [26,27].

The aim of this work was to investigate iodine staining protocols for large samples, such as the meniscus, in different animal species. The relevance of the study lies in its detailed evaluation of the CA diffusion mechanism within the meniscus, considering the width of the tissue and the time-dependent changes in radiodensity. Additionally, volume changes caused by the staining processes were evaluated and the iodine ions taken up by the tissue were identified. This comprehensive approach provides valuable insights into optimizing iodine staining protocols, enhancing the visualization of soft tissues, and ultimately improving the accuracy and efficacy of micro-CT imaging.

## 2 Materials and methods

### 2.1 Sample fixation and staining procedure

**Fig. 1:**
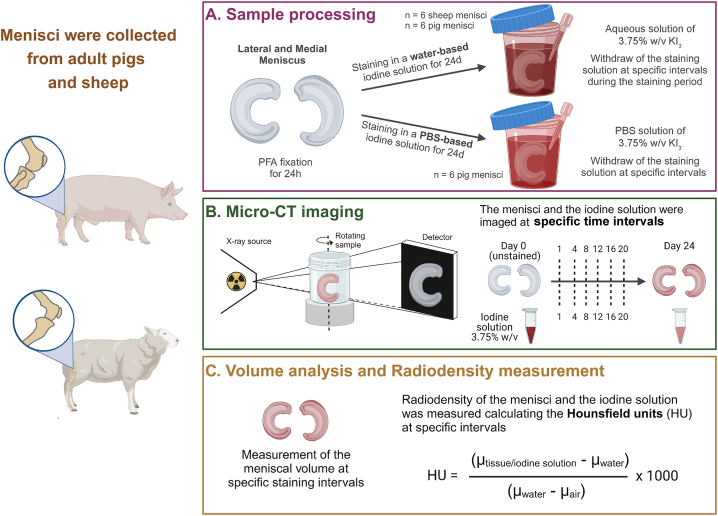
**Schematic illustration of the materials and methods applied in this study.** (**A**) Sample processing steps. (**B**) Micro-CT imaging analysis. (**C**) Volume analyses and radiodensity measurements.

Three Swiss alpine sheep (*Ovis aries*) and six mix breed pig (*Sus domestica*) hindlimbs were obtained from respectively two and three animals (Musculoskeletal Research Unit – MSRU -, University of Zurich, Switzerland). Review and/or approval by an ethics committee was not required for this study because the hindlimbs were collected from research animals after they were sacrificed at the study-specific endpoints of other research projects. The knee joints were freed from surrounding soft tissues. The patella as well as the collateral ligaments were excised to expose both menisci. Then, sheep menisci (n = 3 medial and n = 3 lateral) and pig menisci (n = 6 medial and n = 6 lateral) were then isolated from the knee joint capsule and cut at the level of the bony attachments of the anterior and posterior horns. The entire menisci were fixed for 24 h in 4 % paraformaldehyde (PFA) and then immersed for 48 h in either water or PBS according to the subsequent staining solution. All the sheep and six pig medial and lateral menisci were soaked in an aqueous solution of Lugol (KI_3_) (i.e., 1.25 % w/v of I_2_ and 2.5 % w/v of KI; total CA volume for each sample ca. 100 ml) for a total of 24 days. The remaining six pig medial and lateral menisci were stained for the same duration in a phosphate-buffered saline (PBS)-based KI_3_ solution with the same concentration (total CA volume for each sample ca. 100 ml). Aliquots of 1 ml of the staining solutions were collected at 8 time points up to 24 days and stored for the subsequent analyses with micro-CT, Ultraviolet–visible (UV–vis) spectroscopy, and pH measurements (Fig. 1A).

### 2.2 Micro-CT imaging

The menisci (wrapped in parafilm), as well as 1 ml samples from the staining solution were scanned with micro-CT (EasyTom XL Ultra 230–160 micro/nano-CT scanner, RX Solutions, Chavanod, France) at specific time points: before staining (day 0), and after 1, 4, 8, 12, 16, 20, and 24 days of immersion in staining solution (Fig. 1B). The images were acquired by setting a rotation step of 0.25°, a number of average frames of 3, and 5 images per frame. The scanner operated at 70 kV and 70 μA, with a nominal resolution set to 25 μm or 30 μm depending on sample size, and each acquisition took 14 min. For higher-resolution imaging, scans were conducted at 8.5 μm and 2.5 μm voxel sizes. The parameters for the 8.5 μm scans included a rotation step of 0.3°, a frame rate of 1.5, an average of 5 frames per scan, and acquisition time of 64 min. For the 2.5 μm scans, the scanner was operated at a voltage of 90 kV and a current of 60 μA, with a frame rate of 1, an average of 5 frames per scan, and a rotation step of 0.18°. In this case, the acquisition time was 168 min. All the CT datasets were reconstructed using the filtered back-projection algorithm, a small ring artifact reduction and a 75 % Sinus window function.

### 2.3 Data processing

The CT datasets were analyzed using the open-source image processing package Fiji and the software application Avizo (Thermo Fisher Scientific, MA, USA). The meniscal tissue was segmented using the IsoData algorithm and for each sample, the segmented volumes were normalized and calculated at specific staining time points (Fig. 1C). The distribution of CA in the tissue was visualized creating 3D volume renderings using the maximum intensity projection (MIP) technique and averaging 150 spatially consecutive radial slices of representative menisci for each time point.

The original CT datasets were converted into an 8-bit format, and the linear attenuation coefficient (μ) was measured for each sample at specific staining times. To provide a quantitative standardize measure of the tissue radiodensity, the Hounsfield Unit (HU) values were calculated for each meniscal sample at different time points during the staining process.

**Fig. 2:**
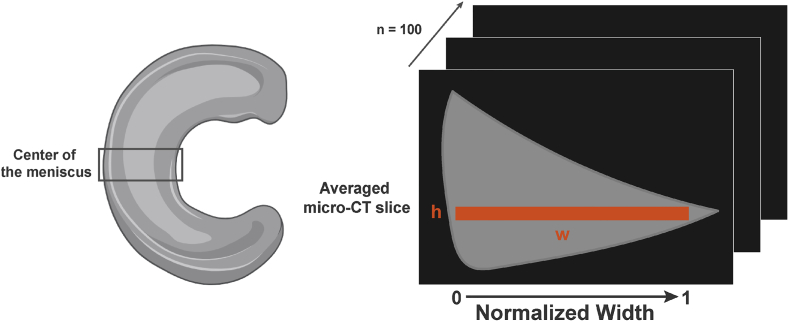
**Schematic illustration of the analyses of contrast agent uptake over the width of the menisci.** Contrast agent uptakes were determined within a region of interest ROI, averaged from 100 CT slices taken from anatomical radial slices of the center of the meniscus.

To determine the CA distribution along the radial direction from the outside of the meniscus to the inside, CT data were analyzed using MATLAB (R2019a, MathWorks, Inc., Natick, MA, USA) at different time points. CA uptakes were determined within a region of interest (ROI) extending from the outer periphery to the inner regions of the meniscus, with radial width dimensions varying depending on the species and sample and a fixed height of 0.25 mm. The values were averaged from 100 CT slices taken from anatomical radial slices of the center of the meniscus (Fig. 2). The gray levels of the images were converted to HU, and the uptake of CA within the meniscus, expressed as a normalized percentage, was determined by subtracting the initial images without CA from the images with CA and normalizing with the mean of the maximum HU value in the images. The formula used to calculate the normalized uptake in % was:

$$U\%=\frac{\left(HU-{HU}_{day0}\right)}{\left({HU}_{\max}-{HU}_{day0}\right)}\times 100$$

where U% is the percentage of the contrast agent uptake, HU is the Hounsfield Unit value of the current image, HU_day0_ is the mean HU value of the day 0 image (baseline without contrast agent), and HU_max_ is the maximum HU value observed across all images. Then, the uptake along the width of the meniscus was then calculated by averaging the HU values across columns (i.e., the 0.25 mm height) for each image, resulting in a one-dimensional uptake profile parallel to the radial axis of the central meniscus. These profiles were combined across all time points and samples to calculate a mean uptake profile. The width of the menisci was normalized from 0 to 1 to facilitate comparison. The resulting profiles were visualized using a heatmap with a color scale from blue to red through green and yellow representing the uptake levels over time and depth.

The staining solutions were segmented utilizing the software application Avizo (Thermo Fisher Scientific, MA, USA). Similarly to the radiodensity measurement of the menisci, the original CT datasets were converted into an 8-bit format, and μ was measured for each sample (Fig. 1C). To provide a quantitative standardize measure of the solution radiodensity, the HU values were calculated for each iodine solution at specific time points during the staining process. The percentage change in radiodensity was then determined by comparing the HU values from day 0 to day 1 of the staining solutions:

$$R\%=\frac{\left({HU}_{day0}-{HU}_{day1}\right)}{\left({HU}_{day0}\right)}\times 100$$

### 2.4 UV–visible spectroscopy and pH analysis of the staining solutions

Staining solutions were diluted by a factor of 500 and the absorbance spectra were acquired with 1.5 nm resolution and a scan rate of 300 nm/min in the range of 210–800 nm using a spectrophotometer (Varian Cary 50 UV–vis, Varian, Inc., California, USA). Three distinct peaks at 228, 288, and 351 nm, corresponding to the iodine ions, were identified in the Lugol solution. To investigate the uptake of iodine ions by meniscal tissue, the water- and PBS-based Lugol solutions were analyzed at days 0, 1, and 24. Before sample analysis, a solvent baseline measurement, either water or PBS, was recorded for use as a blank sample. The samples were diluted as follows: the water and PBS phases were diluted 3000 times to measure I^−^ and 500 times to measure I_3_^−^.

Regarding the pH analysis, pH values of both water- and PBS-based iodine solutions were measured at days 0, 1, and 24 of the staining period using a FiveEasy pH meter (Mettler-Toledo, Schweiz GmbH).

### 2.5 Statistical analysis

Single Factor Anova and Tukey-Kramer's Test were performed for multiple comparisons. Statistical significance was set at *P* < 0.05.

## 3 Results

### 3.1 Micro-CT imaging of the menisci during the staining period

**Fig. 3:**
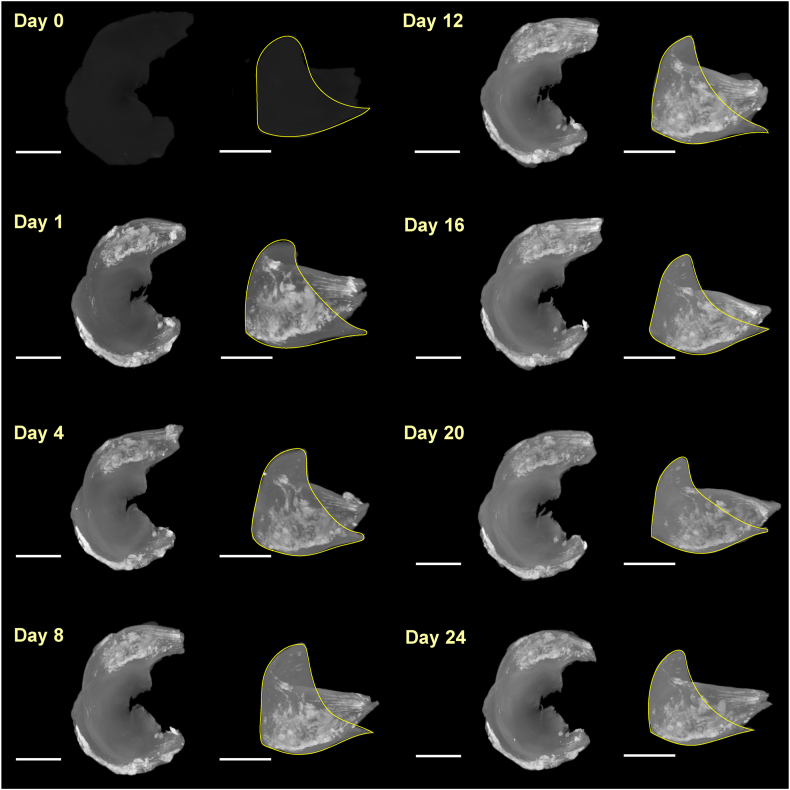
**Micro-CT imaging of the menisci during the staining period.** Axial and radial 3D volume renderings of a sheep meniscus during the staining period using the maximum intensity projection (MIP) technique. The yellow line highlights the triangular shape of the meniscus in the radial orientation. Scale bars in the axial views: 10 mm; scale bars in the radial views: 5 mm.

Following the automatic segmentation approach described above, 3D volume renderings of the tissue were generated for each staining time point. The 3D renderings captured temporal changes in volume, in radiodensity and in dyeing of different structural features (Fig. 3).

**Fig. 4:**
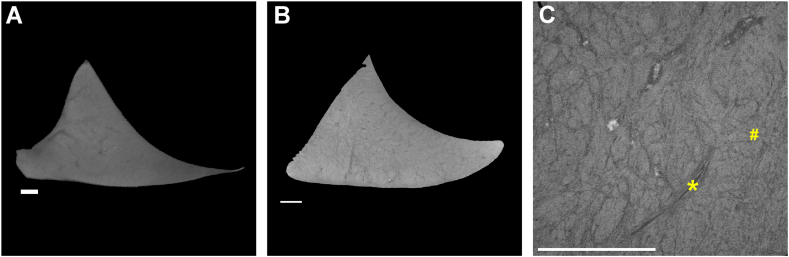
**Cross-sectional 2D images of a stained meniscus scanned at different resolutions after 24 days of staining.** The menisci were imaged at nominal resolutions of 25 μm (**A**), 8.5 μm (**B**), and high-resolution of 2.5 μm (**C**). Blood vessels are indicated with an asterisk (∗), and collagen fiber organization is highlighted with a hash symbol (#). Scale bars: 1 mm.

At day 24 of the staining period, the menisci were imaged at progressively higher resolutions, i.e. 25 μm (Fig. 4A), 8.5 μm (Figs. 4B) and 2.5 μm voxel size (Fig. 4C). The higher resolution scan (Fig. 4C) allowed the visualization of distinct anatomical features of the meniscus, including collagen fiber orientation and blood vessels. This highlights the efficacy of the staining process in enhancing the visibility of specific anatomical and structural characteristics.

### 3.2 Volumetric measurement of the menisci

**Fig. 5:**
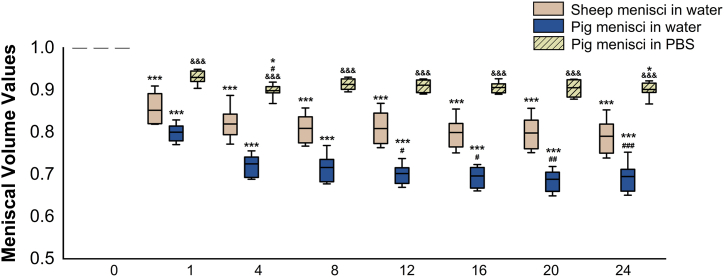
**Volumetric measurement of the menisci during the staining period.** Boxplots are coloured in beige for sheep menisci and in blue for pig menisci stained in water-based iodine solution, and in light green for pig menisci stained in PBS-based iodine solution. For each sample group: day 0 *vs.* remaining staining times ∗∗∗ 0.001 < *P*, ∗ 0.01 < *P* < 0.05; day 1 *vs.* remaining staining times ### 0.001 < *P*, ## 0.001 < *P* < 0.01, # 0.01 < *P* < 0.05; day X of pig menisci in PBS *vs.* corresponding day X of pig menisci in water &&& 0.001 < *P*.

Volumetric analyses were performed on all samples. Immersion in the CA solution caused a reduction in tissue volume. Using the initial volume (day 0) as the reference, one day of staining resulted in a statistically significant average reduction in meniscal volumes: 15 % for sheep and 20 % for pigs when stained in water-based solutions. The volume shrinkage remained consistent over the remaining staining period. Specifically, by days 4 and 24, sheep samples showed volume reductions of 18 % and 21 %, respectively, while the volumes of pig samples decreased of 27 % on day 4 and 30 % on day 24 (Fig. 5).

A decrease in volume was also observed in pig meniscal samples stained in the PBS-based iodine solution. However, this decrease was statistically lower than in those stained with the water-based solution and did not progress beyond day 4. Specifically, the volumetric decreases for days 1, 4, and 24 were 7 %, 10 %, and 10 %, respectively, compared to day 0 (Fig. 5).

### 3.3 Radiodensity measurement of the menisci

**Fig. 6:**
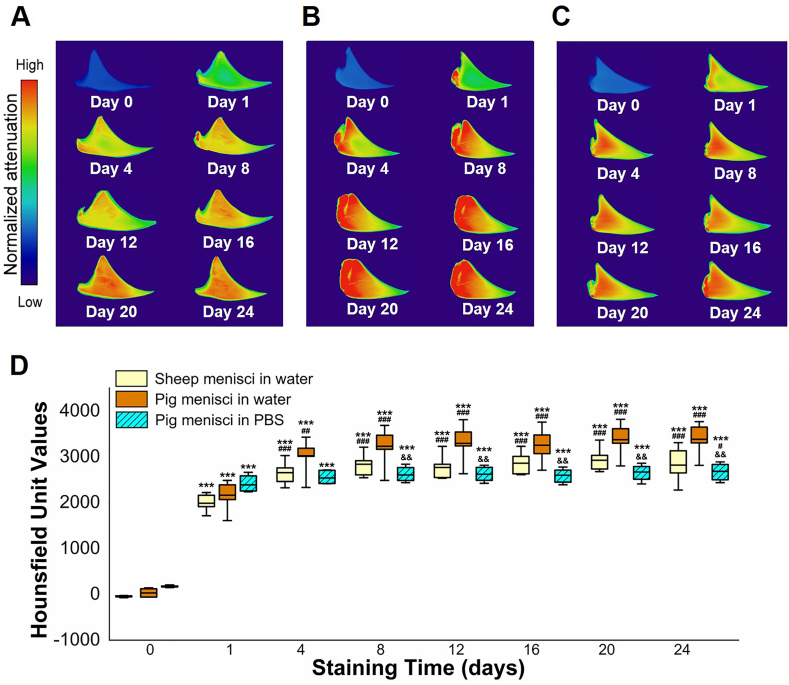
Radiodensity measurement of the menisci during the staining period. Radial micro-CT images (average of 150 consecutive slices) of sheep (**A**) and pig (**B**) samples in water and pig (**C**) samples in PBS for all time points. (**D**) HU values of each sample group for all time points. Boxplots are coloured in yellow and in orange for sheep and pig menisci stained in water-based iodine solution and in light blue for pig menisci stained in PBS-based iodine solution. For each sample: day 0 *vs.* remaining staining times ∗∗∗ 0.001 < *P*; day 1 *vs.* remaining staining times ### 0.001 < *P*, ## 0.001 < *P* < 0.01, # 0.01 < *P* < 0.05; day X of pig menisci in PBS *vs.* corresponding day X of pig menisci in water && 0.001 < *P* < 0.01.

Radial micro-CT images of sheep (Fig. 6A) and pig menisci (Fig. 6B and C) were captured throughout the staining period of 24 days. These images suggested that the water- and PBS-based iodine solutions primarily diffused through the peripheral region of the tissue, while the inner region exhibited limited uptake. The low attenuation at the exact surfaces is an artifact due to anatomical misalignment of the overlapping surfaces across the 150 slices. Radiodensity analyses of the menisci stained in the water-based iodine solution revealed an increase in HU over the first four days of the staining period (Fig. 6D). In sheep and pig samples, HU values from day 8 until day 24 are statistically significant higher compared to day 0 (sheep average value: 49 ± 17.3 HU, pig average value: 28 ± 96.7 HU) and day 1 (sheep average value: 1987 ± 182.3 HU, pig average value: 2163 ± 321.3 HU). From the radiodensity graph (Fig. 6D), an increasing trend can be observed between days 1 and 4 (sheep average value: 2655 ± 244.3 HU, pig average value: 3024 ± 369.1 HU) in both sheep and pig samples. After this initial staining period, no additional iodine uptake was observed. Average HU values on days 8 and 24 were 2838 ± 240.7 HU and 2820 ± 366.6 HU for sheep menisci, 3231 ± 369.1 HU and 3384 ± 334.1 HU for pig menisci, respectively.

Similarly to the samples stained in iodine water-based solution, HU values of the menisci stained in the PBS solution increased significantly from day 0 (average value: 175 ± 20.5 HU) to all other staining times. After day 1 (average value: 2388 ± 186.0 HU), the radiodensity values remained relatively stable (average value day 24: 2684 ± 192.8 HU) and from day 8 to day 24, the radiodensity values were significantly statistically reduced compared to the corresponding samples stained in the water-based iodine solution (Fig. 6D).

### 3.4 Width-wise contrast agent diffusion within the meniscus

**Fig. 7:**
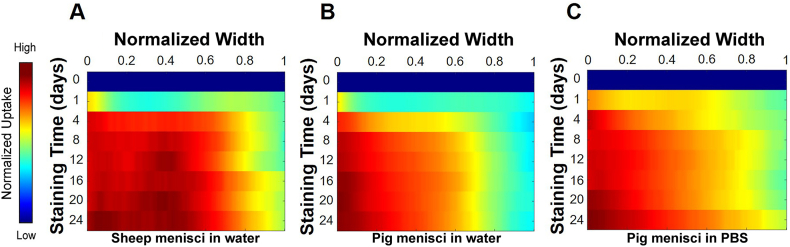
**Width-wise contrast agent diffusion within the meniscus during the staining period.** Mean (n = 100 slices per sample) contrast agent diffusion of sheep menisci (**A**; n = 6 samples) and pig menisci (**B**; n = 6 samples) stained in water-based iodine solution. (**C**) Mean (n = 100 slices per sample) contrast agent diffusion of pig menisci (n = 6 samples) stained in PBS-based iodine solution.

The CA diffused in the three different sample groups solely in one direction, specifically from outwards to inwards (Fig. 7). In sheep menisci stained in the aqueous iodine solution, the uptake of the CA increases significantly during the first 4 days, during which 70 % of the tissue width is stained. However, iodine absorption continued throughout the entire staining period until day 24 (Fig. 7A). For pig menisci stained in the aqueous-based solution, 8 days of staining are necessary for iodine to diffuse into 70 % of the tissue width. However, less diffusion is observed into the remaining inner portion of the tissue (Fig. 7B). The iodine solution in PBS diffused more rapidly than the aqueous counterpart into pig menisci. Indeed, after one day of staining, the iodine uptake is higher in this group of samples compared to those immersed in the aqueous solution. Similarly to the other sample groups, iodine diffusion continues throughout the entire staining period (Fig. 7C).

### 3.5 Radiodensity measurement of the staining solutions

**Fig. 8:**
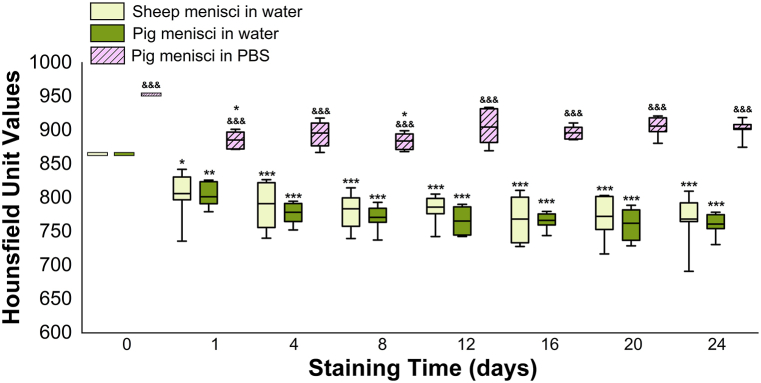
Radiodensity measurement of the contrast solutions during the staining period. HU values of the water-based contrast solutions are outlined in light green for sheep menisci and in dark green for pig menisci, the PBS-based contrast solution is outlined in pink. For each sample group: day 0 *vs.* remaining staining times ∗∗∗ 0.001 < *P*, ∗∗ 0.001 **<***P* < 0.01, ∗ 0.01 < *P* < 0.05; day 1 *vs.* remaining staining times ### 0.001 < *P*, ## 0.001 < *P* < 0.01, # 0.01 < *P* < 0.05; day X of pig menisci in PBS *vs.* corresponding day X of pig menisci in water &&& *P* < 0.001.

As expected, the radiodensity analyses of the iodine solutions exhibited an opposite trend to that of the meniscal samples. Specifically, the greatest decrease in iodine concentration occurs during the first day of staining for both water- and PBS-based solutions (Fig. 8). These data support the previously described radiodensity results of meniscal tissue, which showed a significant increase in iodine uptake after one day of staining (Fig. 6D).

The percentage change in radiodensity, accounting for initial HU differences, showed similar decreases in CA uptake over time for both solutions. Specifically, the decrease from day 0 to day 1 was 6.75 % for sheep menisci in water, 7.29 % for pig menisci in water, and 7.02 % for pig menisci in PBS. These values indicate comparable diffusion behavior of the CA in both water-based and PBS-based solutions.

### 3.6 UV–visible spectroscopy and pH analysis of the staining solutions

**Fig. 9:**
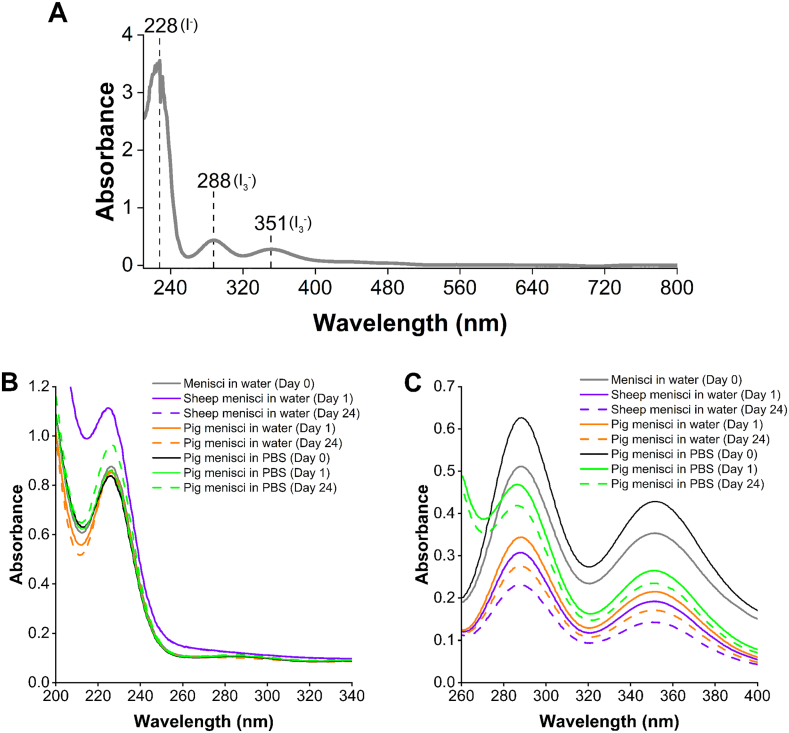
**UV–visible spectroscopy analysis of the iodine solutions.** (**A**) Three distinct peaks were identified at 228, 288, and 351 nm and assigned to I^−^ and I_3_^−^. (**B**) I^−^ was measured at days 0, 1, and 24 of the staining period for each sample group (diluted 3000 times). (**C**) I_3_^−^ was measured at days 0, 1, and 24 of the staining period for each sample group (diluted 500 times).

Lugol's solutions were subsequently analyzed using UV–visible spectroscopy, identifying three peaks attributed to two different iodine ions: I^−^ and I_3_^−^ (Fig. 9A). The peak at 228 nm corresponding to I^−^ showed no change in peak intensity after 1 and 24 days in the water-based solution, while a slight difference was observed in the PBS-based solution (Fig. 9B). Whereas, the peaks corresponding to I_3_^−^ exhibited a temporal decrease in intensity across all three sample groups. Both peaks decreased in intensity during the staining period (from day 0 to days 1 and 24), revealing a selective uptake of the I_3_^−^ ion by the meniscus (Fig. 9C). The behavior of iodine ions in different samples explains the radiodensity values of the staining solutions. Indeed, in sheep samples, a maximum concentration of I^−^ and a minimum concentration of I_3_^−^ resulted in radiodensity values similar to pig samples stained in the water-based solution. Conversely, in PBS samples, a higher concentration of I^−^ on day 24 compared to day 1, and a lower concentration of I_3_^−^ on day 24 compared to day 1, led to similar radiodensity values between days 1 and 24.

The pH values of the water- and PBS-based iodine solutions were measured at the same staining time points as the UV–visible spectroscopy analysis (days 0, 1, and 24). At day 0, the water-based iodine solutions were acidic with a pH of 4.37, while the PBS-based solution had a neutral pH of 7.70 (Table 1). Over the staining period, the pH values of both iodine solutions decreased, with the water-based solutions consistently reaching a more acidic level (Table 1).

**Table 1:** **Comparison of pH values of the iodine solutions.** pH mean values were calculated at days 0. 1, and 24 for each sample group. Data are shown as mean ± SD.

Staining times	Mean pH Values		
	Sheep menisci in water	Pig menisci in water	Pig menisci in PBS
**Day 0**	4.37 ± 0.04	4.37 ± 0.04	7.70 ± 0.16
**Day 1**	4.04 ± 0.03	3.94 ± 0.07	7.20 ± 0.10
**Day 24**	3.50 ± 0.17	3.30 ± 0.28	5.58 ± 0.19

## 4 Discussion

In this study, the diffusion patterns of iodine-based CA solutions into the menisci of sheep and pigs was investigated using 3D imaging. Pig and sheep are the most frequently used animal models for meniscus repair and regeneration due to their similar anatomical features and biochemical characteristics to the human counterpart [28,29]. Micro-CT was employed to visualize and quantify the diffusion of the CA within the tissues at various time points during the staining process. The non-destructive nature of micro-CT permitted to analyze and subsequently visualize each sample at eight different time points (from day 0–24).

The unstained menisci (day 0) provide information on the volume and shape of the samples. However, specific anatomical and structural characteristics were not discernible due to the low inherent contrast of the meniscus, a typical characteristic of soft, low density tissues. Therefore, the use of CAs is necessary for the visualization of these tissues by X-ray-based imaging [14,16,19]. In 2009, Metscher's study was the first one to use and compare different CAs for the visualization of soft tissues using micro-CT [16]. Specifically, he demonstrated how simple staining methods based on iodine and phosphotungstic acid (PTA) enabled high-resolution visualization of embryonic chicken tissues, even permitting the distinction of individual cells [16]. Considering the size of the meniscus and the ease of preparing the solution, Lugol solution (KI_3_) was used to contrast sheep and pig meniscal samples for a total period of 24 days. Previous studies have shown that potassium iodide solution is easy to prepare and allows staining even large samples [13,15,30]. The work of Pauwels et al. involved the study of various chemicals (n = 12) that could be used as CAs to stain mice paws by immersion of the samples for contrast-enhanced micro-CT [13]. After 24 h of immersion, only the iodine-based and sodium tungstate solutions were able to penetrate the samples entirely, proving their effectiveness as CAs for larger specimens [13]. However, additional days of staining were necessary in this study to permit the diffusion of the CA throughout the entire sample, probably due to the larger size of the sheep and pig menisci compared to mice paws. This is in line with the work of Disney et al., were completely staining a quarter segment of a bovine intervertebral disc (IVD), a fibrocartilagineous tissue with a composition similar to that of the meniscus, required 14 days of incubation in KI_3_ [31].

The volumes of all samples were calculated on days 0, 1, 4, 8, 12, 16, 20, and 24 of staining and normalized to the initial volume, in order to allow a more reliable comparison between the various samples. After one day of staining, the volume of the sample reduced in all groups, namely the sheep and pig menisci immersed in the water-based iodine solution as well as those of pigs immersed in the PBS-based iodine solution, by respectively 15 %, 20 %, and 7 %. For the menisci stained in the water-based solution, the volumes also decreased in the subsequent days of staining, albeit less substantially. Whereas for the samples in PBS-based iodine solution, the volume decrease was statistically lower than in those stained with the water-based solution and did not progress beyond day 4. This difference can be attributed to the pH-buffered of the PBS-iodine solution. Indeed, Dawood et al. demonstrated that tissue shrinkage is influenced by the pH of the iodine solutions, with more acidic pH levels resulting in greater shrinkage of the tissue samples [32]. The shrinkage caused by the iodine solutions has also been described by other groups [31,33]. Vickerton et al. demonstrated that the macroscopic changes in the tissue depend on the concentration of the KI_3_ solution [33]. For this reason, in our study, we used the minimum concentration required to achieve adequate contrast of the tissue, i.e. 3.75 %.

In addition, radiodensity, a parameter directly related to the uptake of the CA, was calculated in terms of HU for each meniscal sample throughout the staining process. Traditional diffusion studies often rely on fluorescent or radioactive signals, properties that iodine do not possess. Despite this limitation, iodine-based CAs remain among the most commonly used CAs for micro-CT imaging of biological tissues. Although semi-quantitative, analyses of HU values in tissues provide valuable insights into iodine diffusion within the meniscus. In this study, the HU values of the samples stained with the water-based iodine solution enhanced during the first 4 days of staining, with a greater increase on the first day. In the subsequent period, the tissue seems to cease absorbing the CA. However, a qualitative analysis based on the visualization of micro-CT images was necessary to define a staining protocol and elucidate the diffusion of iodine in the tissue. In fact, as shown in Fig. 6A and B, the contrast continued to diffuse throughout the staining period and its diffusion occurred mainly from the outer portion of the tissue (Fig. 7A and B). Similar results were observed in the images of the samples immersed in the PBS-based iodine solution (Fig. 6, Fig. 7C), while regarding the radiodensity of the tissue, it increased after the first day of staining and subsequently remained stable.

To confirm the radiodensity uptake of the samples, the staining solutions were also analyzed with micro-CT at each time point. Through this analysis, it was observed that an increase in the HU values of the meniscus corresponds to a decrease in the equivalent HU value of the contrast solution, which is to be explained by the diffusion of iodine from the solution to the sample. Degradation over time of the iodine in solution may thus be excluded. Further analyses involved the identification of iodine ions present in the solution and how their concentration differentially evolves during the staining period and in different solutions. For this, UV–visible spectroscopy was used, allowing the identification of three peaks – assigned to I^−^ and I_3_^−^ - present in both water-based and PBS-based solutions [34,35]. The absorbance of I^−^ and I_3_^−^ during the staining period, which is directly proportional to the concentration, highlights a variability in the uptake and retention of I^−^ ions and a selective uptake of I_3_^−^ ions by the meniscus tissue.

Previous studies, such as that of Lakin et al. demonstrated through contrast-enhanced micro-CT and histological analysis that the diffusion of anionic CAs, such as iodine-based ones, is impaired by a high concentration of glycosaminoglycans (GAGs), while it is favored for cationic CAs [36]. Honkanen et al. [25] observed differential uptake of iodinated contrast agents between cartilage and meniscus, noting a higher uptake in the meniscus, which they attributed to its lower GAGs content. In fact, GAGs are less concentrated in the meniscus than in hyaline cartilage, constituting approximately 10 % of the GAG content found in cartilage [37,38]. These previous studies considered either large, iodine-based molecules (such as ioxaglate) or NaI solutions, resulting primarily in I⁻ ions. In our study, however, we found that the majority of the uptake was due to I₃⁻, whose formation requires the presence of I₂, which was absent in the previous experiments [25,36]. We observed a significant increase in HU values, a finding not fully explained by factors related solely to the charge affinity between CA and FDC in tissues, since the negative charge of GAGs generally repels anionic contrast agents.

We therefore hypothesize that the combination between the specific structure and composition of the meniscus and of the triiodide ion (I₃⁻) influences the diffusion and binding behavior of the anionic iodinated CA, extending beyond the simple electrostatic repulsion associated with fixed charge density (FCD). The linear structure of the triiodide ion makes it more likely to interact with regions that offer spatial stability, such as the cavities formed by biochemical components like collagen, proteoglycans, and matrix glycoproteins, which serve as temporary retention sites for I₃⁻ [34,35]. In addition, the presence of polar functional groups, such as -OH and -NH groups from collagen and other extracellular matrix components, facilitates weak hydrogen bonding with I₃⁻. For example, collagen, a protein with amino (-NH₂) and carboxyl (-COOH) groups, exhibits internal polarization at physiological pH, which promotes weak interactions with I₃⁻. Similarly, proteoglycans, although negatively charged due to sulfate (-SO₃⁻) and carboxyl (-COO⁻) groups, contain polar groups capable of forming hydrogen bonds with I₃⁻. Matrix glycoproteins, although less polar and less negatively charged, also possess core protein structures linked to branched chains, providing potential sites for I₃⁻ interactions.

Together, these weak interactions contribute to the unexpectedly high HU values observed in our study. This suggests that the uptake of Lugol's solution into the meniscus is influenced not only by electrostatic factors, but also by the unique structure, size, and binding capabilities of the I₃⁻ ion, allowing for localized partitioning with attenuation levels exceeding those of the initial solution, as observed (Fig. 6, Fig. 8).

The influence of the negative FCD induced by GAGs is nevertheless evident in the generally lower uptake in areas with higher GAG content. The inner zone of sheep and pig menisci is richer in GAGs, enhancing the ability of the tissue to withstand compressive loads, while the outer zone has a lower GAG content [39,40]. This different spatial distribution of GAGs in meniscal tissue could explain the greater diffusion of iodine from the outer zone of the meniscus found in our study. The differential distribution of iodine in the tissue can also be explained by the higher vascularization of the external regions compared to the inner areas of the tissue [7,11]. Blood vessels store glycogen, the molecule to which iodine has an affinity, in the vascular smooth muscle cells (VSMCs) of the artery and vein wall [41].

Another important factor to consider is pH, which can have a significant effect on hydrogen bonding. pH influences the ionization state of functional groups involved in bonding (such as -OH, -NH₂, and -COOH), thereby affecting the strength and stability of hydrogen bonds. In our study, we measured a pH at day 0 of 4.37 ± 0.04 for the CA in water and 7.70 ± 0.16 for the CA in PBS. At a neutral pH, as in our CA solution in PBS, the amino (-NH₂) and carboxyl (-COOH) groups in collagen are in their more stable physiological forms, allowing collagen to form stronger and more durable hydrogen bonds. At an acidic pH (such as that observed for CA in water), carboxyl groups (-COO⁻) tend to become protonated, reducing the number of sites available for hydrogen bonding due to reduced partial charges and polar regions. We also observed a pH decrease of 1–2 units over time in all groups (Table 1), likely due to the gradual acidification of Lugol's solution and the subsequent release of paraformaldehyde from tissue fixation [32]. This additional acidification should, therefore, be taken into consideration when defining the optimal staining duration.

This study is not without limitations. First, we did not perform a power analysis prior to data collection; however, we selected a sample size of 6 per group, which is consistent with standard practice for this type of analysis in similar studies [31,42]. The comparative analysis of menisci was based on two animal species, but we took into account that sheep and pigs are the animals with the greatest similarities to the human equivalent and are therefore most commonly used for studies of meniscal regeneration and repair. Furthermore, the use of CAs can cause artifacts in the original structure of the tissue. In fact, in our study, the use of iodine-based solutions caused a volume shrinkage. However, the routine sample processing steps of other imaging techniques (e.g., histology processing protocols), can also cause tissue artifacts, severely damaging the sample [[43], [44], [45]] or failing to achieve maximal resolution obtainable with micro-CT [46]. This potential for shrinkage due to iodine staining may affect imaging accuracy by complicating volume assessment. In addition, variations in meniscal tissue composition may result in inconsistent staining responses. Future studies using multimodal imaging may provide insights to better account for these effects. Given the significant iodine uptake within the first day of staining, future studies would also benefit from including a 12-h time point to capture early diffusion dynamics. While our study focused on longer staining periods based on the extended time required for iodine penetration in large fibrocartilaginous samples [31], the lack of a time point before 24 h may be considered a limitation. Future research could also explore the impact of iodine uptake on the biomechanical properties of the meniscus, testing the same sample imaged with micro-CT, for example, through nanoindentation. However, in such cases, additional considerations should take into account the effects of fixatives, such as formalin used in the present study, which is known to cause cross-linking and increase tissue stiffness [47,48]. This highlights the potential need for contrast agent protocols without chemical fixation, with appropriate adjustments to account for altered diffusion dynamics and potential changes in tissue properties.

In conclusion, this study demonstrated the utility of iodine-based CAs and advanced 3D imaging techniques for visualizing large soft tissue and investigated the iodine diffusion patterns within the meniscal tissue of sheep and pigs, with a particular focus on the mechanism significance of the presence of I₃⁻ ions in enhancing contrast. The non-destructive nature of micro-CT allowed a detailed spatial and temporal analysis, revealing a preferential iodine diffusion through the peripheral region of the meniscus during the staining period. For the sheep samples in the aqueous solution, 4 days of staining are sufficient for iodine to diffuse through 70 % of the sample's width. Whereas, for the pig samples, 8 days of staining in either water- or PBS-based iodine solutions are necessary to reach the same level of diffusion. Therefore, we recommend an 8-day staining period, as by this point, iodine has diffused through at least 70 % of the tissue width in all sample groups, and any additional shrinkage is statistically minimal after the first day of staining. Extending staining time beyond 8 days does not significantly affect tissue shrinkage and radiodensity, but can increase iodine leakage risk, particularly in water-based solutions, as highlighted by Hildebrand et al. and Boix-Lemonche et al. [49,50]. Therefore, an 8-day staining period effectively balances iodine diffusion and stability within the tissue. The UV–vis analysis of the iodine solutions highlighted the differential absorption of iodine ions by the tissue. The findings of this study have potential important implications for the use of iodine-based CAs in imaging studies of the meniscus and offer valuable insights into the diffusion patterns of iodine solutions in the tissue. Moreover, the iodine staining method used in this study enabled detailed visualization of key structural components within the meniscal tissue, particularly collagen fibers and blood vessels, when scanned at high resolution. Identifying these elements is essential for advancing research on meniscal health and injury, with a focus on structural integrity and functionality.

## CRediT authorship contribution statement

**Federica Orellana:** Writing – original draft, Visualization, Methodology, Investigation, Formal analysis, Data curation. **Alberto Grassi:** Writing – review & editing, Investigation. **Katja M. Nuss:** Writing – review & editing, Resources. **Peter Wahl:** Writing – review & editing. **Antonia Neels:** Writing – review & editing, Resources. **Stefano Zaffagnini:** Writing – review & editing. **Annapaola Parrilli:** Writing – review & editing, Writing – original draft, Supervision, Methodology, Funding acquisition, Conceptualization.

## Data availability

The data that support the findings of this study are available from the corresponding author upon reasonable request.

## Funding

The research was supported by the Swiss National Science Foundation (grant no. 197928).

## Declaration of competing interest

The authors declare that they have no known competing financial interests or personal relationships that could have appeared to influence the work reported in this paper.
